# Alliance with EPR Effect: Combined Strategies to Improve the EPR Effect in the Tumor Microenvironment

**DOI:** 10.7150/thno.37198

**Published:** 2019-10-17

**Authors:** Jooho Park, Yongwhan Choi, Hyeyoun Chang, Wooram Um, Ju Hee Ryu, Ick Chan Kwon

**Affiliations:** 1 Center for Theragnosis, Biomedical Research Institute, Korea Institute of Science and Technology (KIST), Hwarang-ro 14-gil 5, Seongbuk-gu, Seoul 02792, Republic of Korea; 2 KU-KIST Graduate School of Converging Science and Technology, Korea University, 145 Anam-ro, Seongbuk-gu, Seoul 02841, Republic of Korea; 3 Department of Cancer Biology, Dana-Farber Cancer Institute, 450 Brookline Avenue, Boston, Massachusetts 02215, United States

**Keywords:** EPR effect, targeted therapy, nanoparticle, cancer treatment, drug delivery

## Abstract

The use of nanomedicine for cancer treatment takes advantage of its preferential accumulation in tumors owing to the enhanced permeability and retention (EPR) effect. The development of cancer nanomedicine has promised highly effective treatment options unprecedented by standard therapeutics. However, the therapeutic efficacy of passively targeted nanomedicine is not always satisfactory because it is largely influenced by the heterogeneity of the intensity of the EPR effect exhibited within a tumor, at different stages of a tumor, and among individual tumors. In addition, limited data on EPR effectiveness in human hinders further clinical translation of nanomedicine. This unsatisfactory therapeutic outcome in mice and humans necessitates novel approaches to improve the EPR effect. This review focuses on current attempts at overcoming the limitations of traditional EPR-dependent nanomedicine by incorporating supplementary strategies, such as additional molecular targeting, physical alteration, or physiological remodeling of the tumor microenvironment. This review will provide valuable insight to researchers who seek to overcome the limitations of relying on the EPR effect alone in cancer nanomedicine and go “beyond the EPR effect”.

## 1. Introduction

The targeted delivery of therapeutics for cancer treatment has been developed alongside the progress of nanotechnology 1, 2. Nanoparticles (NPs) that incorporate one or more cancer drugs, such as chemotherapeutic agents, molecularly targeted drugs, and small interfering RNAs, are intravenously administered for delivery to tumors 3. They are designed to prolong systemic circulation, minimize any off-target interactions, and improve the target-specific accumulation of therapeutics. Although the surface of NPs may be embellished with specific tumor-targeting molecules to induce more “active” targeting, the majority of the current targeting strategies is “passive”, meaning that delivery relies solely on the unique physical characteristics of NPs and tumors 4-6. When tumors form, their rapid growth results in neovasculature with wide fenestrations and suppressed lymphatic drainage 7. Consequently, nanoscale drug carriers that do not readily extravasate into normal healthy tissues effectively pass through the leaky blood vessels of the tumor and accumulate within the target site. This preferential accumulation of NPs in tumors is known as the enhanced permeability and retention (EPR) effect 4, 8. This approach has shown many promising results exceeding those of standard therapeutics, including reduced toxicity in healthy tissues and increased drug concentration in the target site. In fact, multiple nanomedicine carriers, such as liposomes, micelles, albumin NPs, and polymer conjugates, have been approved for the treatment of various cancers over the past 20 years 9.

As the field of cancer nanomedicine rapidly progresses, it has become evident that the therapeutic efficacy of passively targeted nanomedicine is immensely influenced by the heterogeneity of the intensity of the EPR effect within a tumor, at different stages of a tumor, and among individual tumors 10. Tumors are heterogeneous by nature and various studies have shown that the EPR effect within tumors is also highly heterogeneous 11-13. Heterogeneity is present among tumor models of different species, diverse tumor types of the same origin, tumors at different locations in the same patient, and even at different stages of the same tumor during its development 14-16. Within a tumor, variations in the thickness and density of the extracellular matrix (ECM), uneven blood flow distribution, and disproportionate vessel permeability have been shown to affect the heterogeneity of the EPR effect 11, 17. These factors vary substantially among tumors and even within the same tumor over time. In addition, the physicochemical properties of a nanoscale drug carrier, such as size, shape, and elasticity, contribute to the heterogeneity of the EPR effect. Several studies have shown that the size, shape, and elasticity of nanomedicine carriers impacted extravasation from the vessel and retention in the tumor site, resulting in varied therapeutic efficacy 18-20. However, it is critical to mention that our current understanding of the EPR effect is mostly based on animal data, especially from fast-growing xenograft models in mice, which have been used most to explain the EPR effect. Experimental data on EPR effectiveness in patients should be further accumulated for the successful clinical use of nanomedicine.

**Figure 1:**
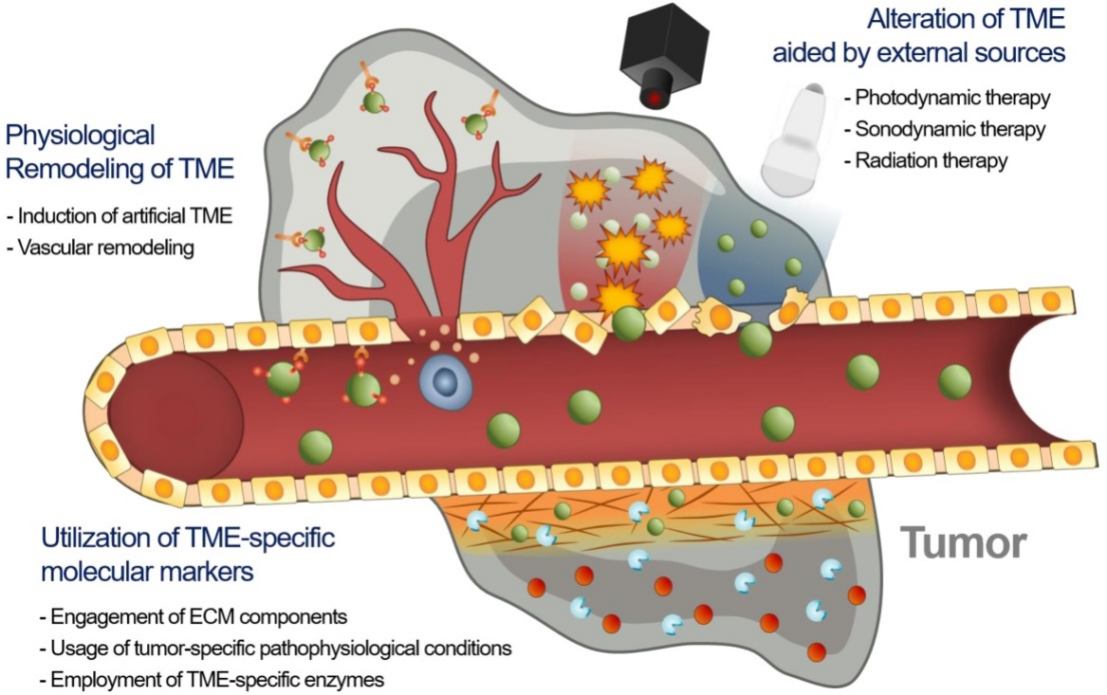
Schematic illustration of three synergistically combined strategies (utilization of TME-specific molecular markers, alteration of TME aided by external sources and physiological remodeling of TME) to improve the EPR effect on the various factors in TME.

Consequently, the delivery of an EPR-dependent nanomedicine can be further enhanced to maximize its therapeutic efficacy 21, 22. Although the benefits of nanomedicine in principle surpass the efficacy of standard therapeutics, its therapeutic efficacy is not always satisfactory because if it is largely influenced by the heterogeneity of the intensity of the EPR effect 23-25. This unsatisfactory therapeutic outcome in mice and humans requires novel approaches to improve the EPR effect by additional different strategies (**Figure 1**). First, tumor microenvironment (TME)-specific molecular markers including ECM components, tumor-specific pathophysiological conditions, and TME-specific enzymes, can be utilized with nanomedicine. Second, physical alteration of TME by photodynamic, sonodynamic, and radiation therapies can be applied to improve the EPR effect. Third, the physiological remodeling of TME aims to improve the EPR effect indirectly by inducing artificial TME or promoting vascular remodeling. In this review, we summarize three different combination strategies based on their enhancement of the EPR effect to maximize the therapeutic benefits of nanomedicine, focusing on the utilization of TME-specific molecular markers, alteration of TME aided by external sources and physiological remodeling of TME.

## 2. Utilization of TME-specific molecular markers

**Figure 2:**
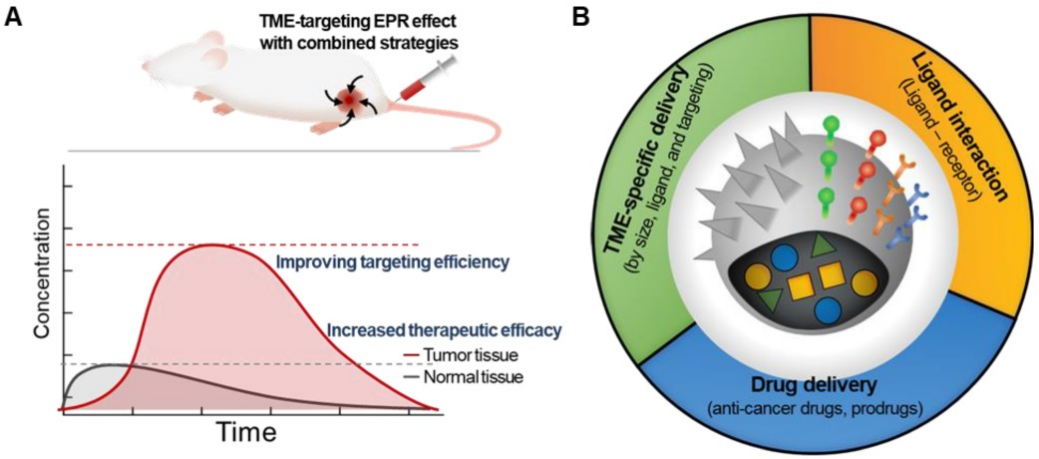
The conventional EPR effect usually resulted in an increased amount of nanomedicine in the tumor tissue. (A) Synergistic strategies can further enhance the accumulation of nanomedicine at the tumor site, indicating improved efficacy. (B) Various attempts to overcome the limitations of traditional EPR-reliant nanomedicine by incorporating additional molecular modification on NPs.

A prominent strategy to enhance the permeation and retention of nanomedicine in tumors, in addition to targeting the tumor cells themselves, is to add a moiety that specifically binds to components of the TME 26. Targeting tumor cells by adding tumor cell-specific binding molecules has been a very popular strategy to overcome the limitations of traditional nanomedicine 27. Since components of TME share many characteristics specific to them, targeting TME with chemical ligands has shown great potential in improving targeting efficiency in drug delivery and enhancing the therapeutic efficacy of cancer treatment **(Figure 2A)** 20, 28, 29. Decorating NPs with TME-targeting ligands is one of the most effective ways to provide nanomedicine with TME-specific targeting ability to overcome tumor heterogeneity, considering that human patients possess remarkably diverse TMEs and that the physiological barriers to macromolecule delivery cannot be simply overcome by using the traditional EPR strategy alone **(Figure 2B)**. The traditional EPR effect of NPs has been limited in clinical use due to unpredictable therapeutic efficiency, low delivery efficacy, and poor clinical outcomes.

### 2.1. Engagement of ECM components

It is clear that the EPR effect is highly associated with the physiological condition and heterogeneity of ECM at the tumor site 30. Potential molecular targets in ECM that could be used to increase the EPR effect exerted on drug delivery systems (DDS) include fibrous ECM proteins, proteoglycan, growth factor receptors, and transmembrane receptors. A dense TME composed of fibrous proteins, such as collagen and fibronectin, directly reduces the vascular transport of nanomedicine 12. Targeting and breaking down these molecules to loosen the ECM facilitates the entry of nanomedicine to the tumor site.

**Figure 3:**
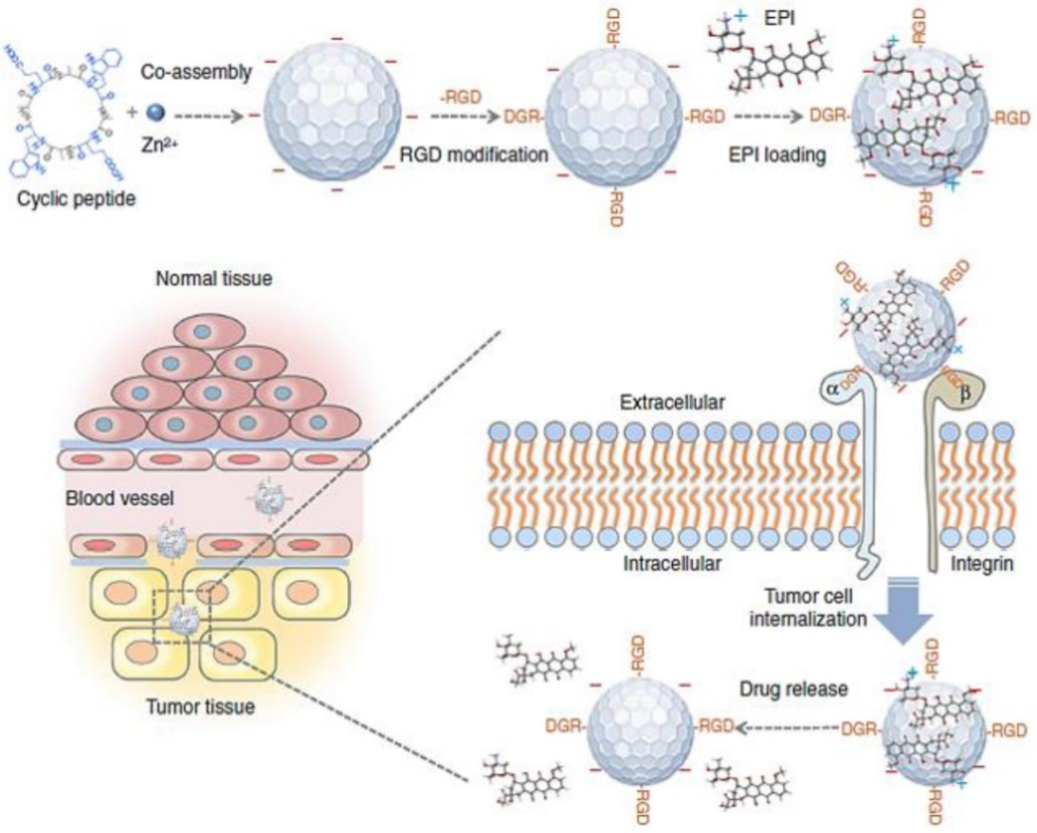
Schematic illustration of coassembled NPs with Zn^2+^ ions, cyclic peptides, epirubicin (EPI) and an RGD moiety show that they can accumulate in the tumor tissue by the EPR effect. Delivery tends to be increased by binding to the overexpressed α_v_β_3_ integrin and tumor cell internalization. Adapted with permission from 35, copyright 2018 Nature Publishing Group.

Among various transmembrane factors, integrin has been well established as a tumor-specific marker of angiogenic activity in ECM 31. Integrin α_v_β_3,_ which is overexpressed on angiogenic vessels, plays a key role in tumor proliferation and metastasis. Targeting integrin α_v_β_3_ leads NPs straight to the angiogenic vessels, increasing the extravasation of the NPs at the tumor site 32. The Arg-Gly-Asp (RGD) peptide can recognize integrin α_v_β_3_ and has therefore been widely used in anti-cancer research to increase the accumulation of macromolecules for the last two decades 33, 34. For example, near-infrared (NIR) fluorescent NPs assembled by multiple cyclic peptides, cyclo[-(D-Ala-L-Glu-D-Ala-L-Trp)_2_-] were modified with RGD moieties and loaded with the chemotherapeutic agent, epirubicin (EPI). They showed increased accumulation in tumor tissue compared to nontumor tissues because of direct binding to integrin α_v_β_3_ in addition to the EPR effect, resulting in enhanced therapeutic efficacy (**Figure 3**) 35. Interestingly, the self-assembled NPs modified with RGD exhibited significantly reduced cardiotoxicity due to the specific delivery to the tumor site. Although RGD-based tumor ECM delivery has great potential to enhance the EPR effect of nanomedicine, some studies have questioned whether RGD-based peptide modification can induce serious toxicity. Indeed, it has been clearly demonstrated that peptide modification with the RGD moiety on NPs could induce severe toxicity associated with immune stimulation 36. Interestingly, this unwanted immune response could be overcome by loading cytotoxic agents in the carrier 36. According to Wang *et al.*, the major immunotoxicity of cyclic RGD peptides in liposomes could not be eliminated by minimizing the amount of the peptide. However, encapsulation of cytotoxic agent (doxorubicin; DOX) shut off the lethal reaction and unwanted immunotoxicity, completely eliminating the unintended lethal IgG response in the body. This result showed the importance of precise control over the formulation of ECM-targeting peptide-based NPs at the development stage to minimize side effects and toxicity. These results appear to have important implications for the clinical application and therapeutic use of peptide modified NPs in the clinic for the optimized EPR effect. Considering the current low clinical use of NPs, these studies serve as valuable references to develop a successful clinically applicable NP with the effective EPR effect for patients.

Receptors that overexpressed in cancer cells, such as epidermal growth factor receptor (EGFR), have been investigated as binding sites for the targeted delivery of anti-cancer drugs. EGFR is often overexpressed in many types of cancers, and is involved in signaling pathways to regulate cell proliferation, differentiation, and inhibition of apoptosis 37. The selection of targeting ligands on the surface of nanomedicine is critical in the design of formulations for EGFR targeting. Most types of ligands used for targeting EGFR have been monoclonal antibodies or fragments thereof, such as cetuximab, trastuzumab or panitumumab. Recently, endogenous ligands such as epidermal growth factor (EGF) have shown great potential for targeting EGFR on tumor cells. Because EGF has a smaller molecular weight (~ 6 kDa) than antibodies, targeting EGFR in TME offers unique advantages: the targeted nanomedicine penetrates more deeply into the tumor, and more rapid *in vivo* clearance can be facilitated 38. Zalba *et al*. developed EGFR-targeted liposomes coupled with EGF for selective delivery of anti-cancer drugs, oxaliplatin into tumors. EGF-conjugated liposomes significantly decreased the IC_50_ of oxaliplatin in EGFR-positive colorectal cancer cell lines without enhancing the cytotoxicity of oxaliplatin in EGFR-negative colorectal cancer cell lines 39. Interestingly, unlike free EGF, EGF coupled on the surface of the liposomes was not able to activate EGFR by EGF-EGFR docking. Therefore, EGFR in TME can be successfully targeted to improve the conventional EPR effect of NPs.

Hyaluronan-based macromolecules that target CD44 receptors have been used to improve the targeting of ECM around tumors and present therapeutic potential in a number of cancers 40. CD44 is often upregulated in various cancer cells and can be a target for hyaluronic acid (HA), which is one of the main components of ECM. HA-based NPs could preferentially accumulate at the tumor site via the EPR effect by binding to CD44. The significance of increasing the EPR effect with additional targeting molecules was clearly shown in a recent study by Liu *et al.* 41. They modulated both the size of HA-based NPs and the loading components to synergistically suppress tumor growth and increase NPs accumulation in tumors. HA-shielded 200 nm NPs displayed an optimal EPR effect in 4T1 mouse breast tumor-bearing mice by achieving enhanced tissue penetration, suppressing the growth of primary tumor by 95%, and inhibiting tumor metastasis by 90%. In addition, Yaqi *et al.* developed a thermosensitive self-assembled nanoplatform that codelivers a matrix metalloproteinase inhibitor (marimastat) and HA-conjugated paclitaxel (PTX) prodrug for dual targeting of the TME and tumor cells. This combination promoted drug accumulation at tumor, tumor growth inhibition (12-fold, compared with the PTX-treated group), and metastasis inhibition (100%, compared with the control group), indicating that the combination of TME-targeting a HA-based nanomedicine with TME modulator is a highly promising strategy for cancer treatment 42. To enhance the EPR effect, HA-based cell-penetrating peptide-modified lipid NPs were also prepared and evaluated in hepatocellular carcinoma cells. These NPs effectively penetrated the ECM and accumulated in the tumor due to the enhanced EPR effect, as demonstrated by low-intensity focused ultrasound (LIFU) imaging. Furthermore, dual targeting strategies with EGFR and CD44 have recently become a focus of research for the EPR effect enhancement 43, 44. Although the dual combination of EGFR and HA targeting has not yet been widely studied, it has appeared as an efficacious way for tumor-targeted therapy to decrease the uncertainty of single targeting. In addition, PEGylated recombinant human hyaluronidase (PEGPH20) has been utilized to eliminate HA in TME. PEGPH20 has shown increased therapeutic effects *in vivo*, inhibiting tumor progression and metastasis. Thus, HA-degradable PEGPH20 has been investigated in combination with anti-cancer agents including gemcitabine, and has shown an increased therapeutic index in animal models 45, 46. It is currently undergoing evaluation in phase III global clinical trial for treatment of pancreatic cancer patients 47.

### 2.2. Usage of tumor-specific pathophysiological conditions

As one of the characteristics of fast-growing malignant tumors, hypoxia is the common result of imbalance between the abnormal blood vessels and the high demand for nutrients and/or oxygen 48. Hypoxia is known to be closely associated with tumor angiogenesis and metastasis, and it can lead to multi-drug resistance (MDR). Nanotechnology has an advantage in treating hypoxic conditions because the unique low oxygen environment provides an opportunity for stimuli-targeting NPs, and the poor angiogenesis can enhance the extravasation of macromolecules in the tumor tissues 23, 49. To increase the EPR effect, NPs can be designed to bind directly to hypoxia-specific molecular markers, to reduce hypoxia-specific gene expression through small interfering RNA (siRNA) treatment, or to release cytotoxic drugs specifically under hypoxic conditions. As a hypoxia-specific molecular marker, increased expression of phosphatidylserine, a phenomenon normally associated with apoptosis, is observed on the external layer of hypoxic tumor cells and tumor-associated endothelial cell membranes. For example, Saposin C, a lysosomal protein that binds to phosphatidylserine, has been utilized to build NPs that bind to the hypoxic TME 50, 51. The resulting NPs showed impressive therapeutic efficacy in the glioblastoma model, crossing the blood-brain barrier, exhibiting specific retention in the tumor tissue, and sensitizing the hypoxic cells.

Another application of nanotechnology that takes advantage of the intratumoral hypoxic conditions is delivering a siRNA that targets a hypoxia-related gene, hypoxia-inducible factor-1α (HIF-1α), which is transcribed into HIF-1α protein. HIF-1α is deeply associated with the activation of a series of genes that aggravate the tumor condition, such as promoting cell proliferation, migration, angiogenesis, low pH, and MDR 52. A cationic micellar NP that incorporates HIF-1α siRNA (NP/siHIF nanocomplex) not only efficiently reduced tumor cell proliferation, migration, and angiogenesis *in vitro*, but also showed tumor growth inhibition and reduced MDR1 gene expression *in vivo* upon systemic administration 53, 54. Perche *et al.* further utilized the hypoxic condition and developed a hypoxia-responsive copolymer that specifically exposes siRNA cargo to hypoxic tumor cells 55. They developed a lipid-conjugated, polyethylene glycol (PEG)-shielded polyethyleneimine (PEI) nanocomplex with a hypoxia-sensitive linker (azobenzene; nitroimidazole derivative) and anti-green fluorescent protein siRNA. Under hypoxic conditions, the azobenzene linker is able to be degraded to release the protective PEG layer, thereby exposing the siRNA to the hypoxic tumor tissue and enabling its hypoxia-dependent cellular uptake into A549 tumor cells (spheroids). PEI is one of the most widely studied and the most successful cationic polymers for delivery of nucleic acid including siRNA. However, high molecular weight PEI has not only exhibited high transfection efficiency but also shown significant systemic toxicity. To reduce the potential toxicity of PEI-based delivery system, PEI has been mixed with other polymers, especially PEG. These various efforts have led to the successful clinical application of PEI to deliver vulnerable genetic biomaterials 56. Similar to the azobenzene linker, 2-nitroimidazole has been widely utilized for hypoxia-sensitive drug delivery. Hydrophobic 2-nitroimidazole is converted to hydrophilic 2-aminoimidazole via a series of reductive reaction under hypoxic conditions. Thambi *et al*. introduced hydrophobic 2-nitroimidazole on the surface of carboxymethyl dextran-based NPs and loaded chemotherapeutic agents, DOX, within NPs 57. The hypoxia-responsive NPs bearing DOX disassembled and released DOX under hypoxic conditions. These NPs demonstrated a high level of tumor-specific accumulation and delayed tumor progression upon systemic administration. Hypoxia-specific nanomedicines allow systemic administration at high doses due to their enhanced therapeutic efficacy and low toxicity. However, the extent of hypoxia varies significantly between tumors and even within a single tumor, and cells experiencing severe hypoxia represent a small population in most solid tumors. To overcome the heterogeneity of hypoxia and achieve the optimal therapeutic effect, artificial induction of hypoxic stress or combination with other existing therapies has also been successfully attempted 58. Stimuli‐responsive DDS provide a great potential for innovative NPs development, triggering a series of synergistic therapeutic effects in the tumor site.

An emerging TME-targeting strategy of utilizing the acidity of TME has been well recognized in designing tumor-specific NPs. Many researchers have considered the acidity of TME as a potential therapeutic target since Otto Heinrich Warburg discovered the pathophysiological acidic condition of tumors as a result of lactate overproduction 59, 60. Therapeutic strategies targeting tumor acidity have demonstrated that it can markedly enhance the tumor-specific accumulation and internalization of macromolecules. The acidic extracellular TME reverses the surface charge of NPs, which can lead to NP aggregation in the TME. Therefore, pH-sensitive NPs would ultimately enhance the preferential retention and accumulation of drugs in tumors by relying on the difference in pH between TME and normal tissues 61-63. Modulation of the pH in TME by NPs and chemical agents can increase the uptake and therapeutic activity of nanomedicines, improving the treatment outcome. For example, treatment of murine 4T1 breast tumor-bearing mice with liposomal DOX and sodium bicarbonate showed 21-fold increase of drug uptake in the tumor site (compared to free nonliposomal bicarbonate) 24 h postinjection 64. These results showed that modulating the acidity of TME holds great potential for conditioning TME towards improved therapeutic activity. These approaches can provide valuable insight into the development of nanomedicine to achieve improved uptake and retention.

### 2.3. Employment of TME-specific enzymes

**Figure 4:**
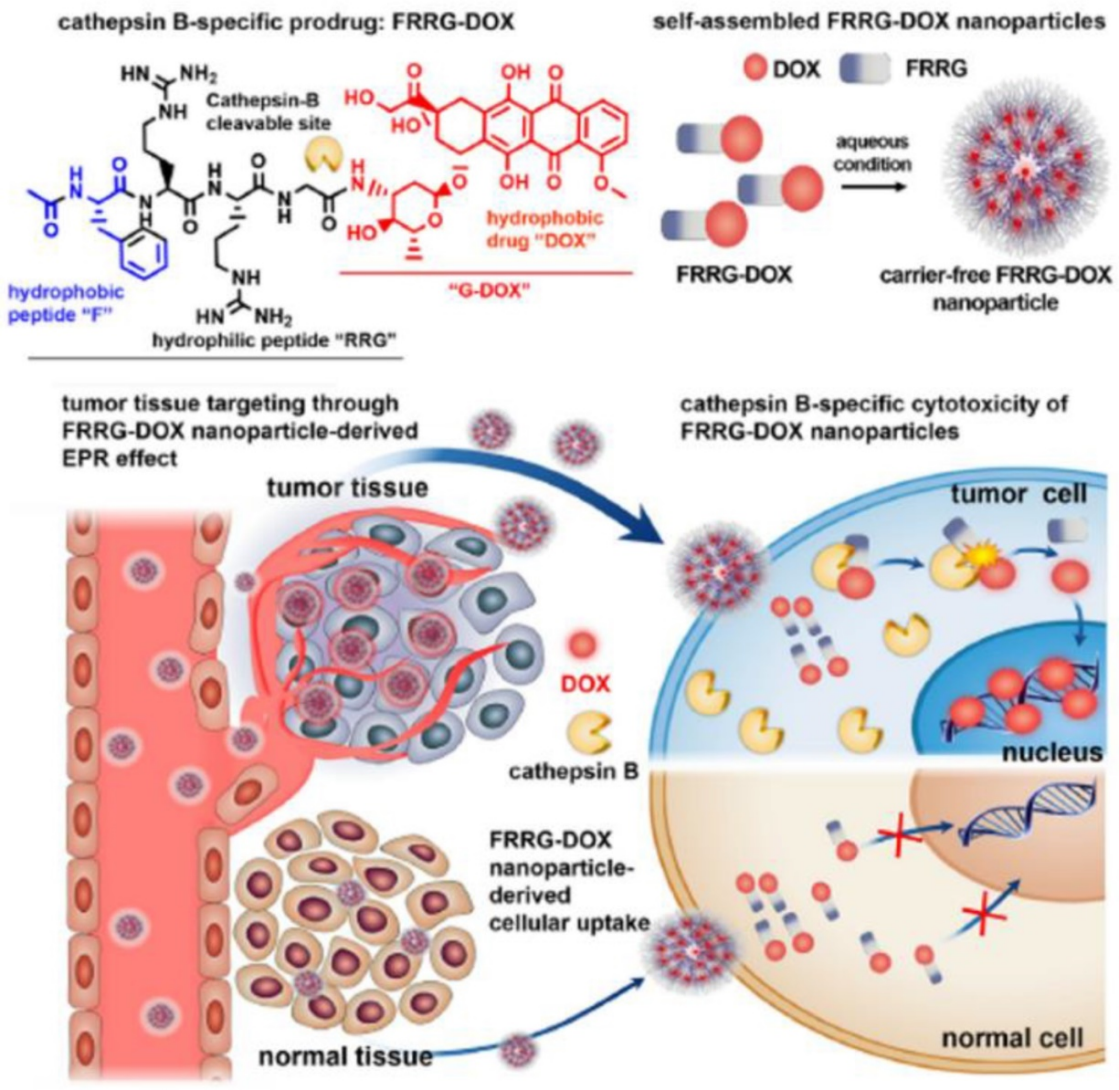
Cathepsin B-specific amphiphilic prodrug (FRRG-DOX; Phe-Arg-Arg-Gly-doxorubicin) that can form a stable NP structure in aqueous conditions accumulates in the tumor site by the EPR effect. The NPs can recover their cytotoxicity by cathepsin B (tumor-specific enzyme) at the tumor site after accumulation, and then show therapeutic effect by the intercalation of DOX into DNA. Adapted with permission from 66, copyright 2019 Elsevier.

TME-specific enzymes can be used in several ways to activate the prodrug in DDS for anti-cancer therapy. This approach has several advantages including tumor-specific accumulation, low systemic toxicity, and high therapeutic efficacy. Enzyme-specific prodrug-based nanomedicine can provide a path forward to enable delivery to tumors for clinical use. The US Food and Drug Administration (FDA) has approved 30 prodrugs during the last decade, which accounts for more than the total number of approved nanomedicine products 65. Additionally, 17% of new chemical FDA-approved drugs have been prodrug types over the past 3 years 65. Clinical challenges and considerations in NPs development can overcome by novel prodrug strategies, solving the current clinical problem of the biopharmaceutical performance of NPs. Prodrug modification may be useful to decrease the critical limitations of NPs in terms of absorption, distribution, metabolism and excretion, which can contribute to their successful clinical application. Implementing a prodrug strategy in early NPs development would accelerate the clinical use and commercialization of nanomedicine. From this point of view, nanoscale self-assembled prodrugs, which exhibit high potential with simple structures and remarkable efficacy, have been extensively evaluated in various recent studies. For example, one of these prodrugs, consisting of peptide and DOX with an overall molecular weight of less than 2000 Da, can self-assemble to form NPs and accumulate in tumors by the EPR effect. It can then be activated by cathepsin B, an enzyme characteristically overexpressed in tumors, releasing free DOX in tumor (**Figure 4**) 66. This approach is important because it can potentially overcome problems NPs currently faced, such as low drug loading efficiency (<10%) and difficulty in mass production. These problems have hindered successful commercialization of novel and functional NPs 67. Most polymer-based nanomedicines with a sound strategy have failed in clinical development due to disappointing efficacy and unknown toxicity. The peptide-based self-assembly strategy would be promising for utilization of the EPR effect in new drug development, considering previously developed polymer-based nanomaterials such as N-(2-hydroxypropyl)methacrylamide-DOX conjugates (PK1 and PK2) showed poor results in clinical trials 68. The programs for developing nanomaterials including PK1 and PK2 were have been largely discontinued. Numerous other complicated polymer-based NPs have also failed in clinical trials due to challenges such as high cost of the manufacturing and unwanted toxicity.

In addition to cathepsin B, various tumor-specific enzymes such as matrix metalloproteinase and caspase, have been utilized to improve the EPR effects of nanomedicines 64, 69. In the case of caspase-3-responsive prodrugs, the induction of apoptosis by external stimuli is initially required to cause the overexpression of caspase-3 in the targeted region of tumor 70. The induced caspase-3 activates the prodrug, and the released anti-cancer drugs further activate the prodrugs by exerting cytotoxic effects on neighboring cancer cells. Interestingly, this repetitive and sequential process -the induction of caspase-3 and activation of the prodrug- propagated the induction of apoptosis and amplified therapeutic effects 70, 71. Similar to the mechanism of enzyme-responsive nanomedicine, glutathione disulfide has been utilized for developing reduction-responsive nanomedicine 72, 73.

Onivyde, a recently approved anti-cancer nanomedicine based on irinotecan can be metabolized to form the active metabolite by serine hydrolase or carboxylesterase. Onivyde has successfully performed in clinical use with improved response rates and therapeutic efficacy 74, 75. In particular, recent findings showed that liposomal irinotecan could completely eliminate the tumors in apoptotic conditions (by radiation therapy) unlike irinotecan itself 76, because it can accumulate in TME and then be metabolized to the active form by tumor-associated macrophages (TAMs). Recent advances further demonstrated that enzyme-triggered supramolecular self-assembly enabled controlled prodrug activation and exhibited increased therapeutic efficacy against cancer cells 77, 78. This self-assembly property of nanomedicine tends to increase the accumulation of anti-cancer drugs in tumor cells. In conclusion, nanotechnology combined with prodrugs and tumor-related enzymes effectively contributes to the enhanced efficacy and safety of nanomedicine, improving the EPR effect.

## 3. Alteration of TME aided by external sources

Alteration of TME aided by external sources such as laser light, ultrasound and radiation in combination with nanomedicine treatment can improve the EPR effect, overcoming the heterogeneous intensity of the EPR effect. Photodynamic therapy (PDT), sonodynamic therapy (SDT) and radiation therapy (RT) can reconfigure the TME by widening vessel leakiness or destroying physical barriers in TME, which can improve drug accumulation and therapeutic efficacy. These approaches have drawn significant attention, due to the universal use against solid tumors and their excellent safety.

### 3.1. Combination with PDT

For the EPR effect, blood vessels of the tumor should be leaky, and NPs should effectively pass through these leaky blood vessels and accumulate within the tumor site. However, vessel leakiness is not sufficient in certain tumors, which results in limited extravasation into tumor tissue and suboptimal EPR effect. Unlike subcutaneous tumor model which have a high level of vessel leakiness caused by rapid tumor development, human tumors are known to have insufficient vessel leakiness for high EPR effects. Therefore, technology to enhance vessel leakiness in tumors is essential for successful clinical translation. A photodynamic stimulus can induce permeabilization of vessels in tumor sites, facilitating the extravasation of NPs 79, 80. After PDT enhances the vessel leakiness in tumor tissue, injected NPs can pass through the leaky blood vessels to accumulate more efficiently within the tumor. In other words, PDT can improve the EPR effect of subsequently administered NPs 67. PDT is considered a safe and minimally invasive therapeutic procedure. Therefore, nanomedicine treatment combined with PDT attracts increasing attention for successful clinical translation.

**Figure 5:**
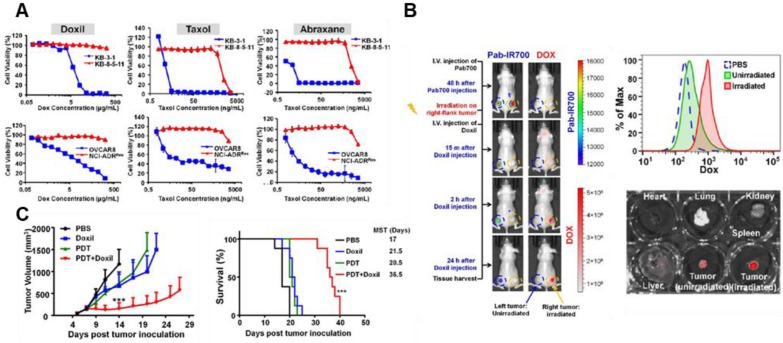
Drug resistance of nanomedicines and PDT (A) Cytotoxicity test (72 h incubation) of Doxil, Taxol, and Abraxane in KB-3-1, KB-8-5-11 and OVCAR8 cells (Pgp negative and chemosensitive) shows that therapeutic efficacy can be greatly limited by Pgp-mediated drug resistance. Red color indicates the Pgp overexpressing cells. (B) Pgp-targeted irradiation with PDT enhances the accumulation of Doxil at the tumor site and its tissue penetration. (C) Tumor growth test in xenograft-bearing mice shows that the combination of Doxil and PDT markedly outperforms the two single treatments in showing improved therapeutic efficacy. Data are presented as the mean ± SD (n = 8, ***p < 0.001). Adapted with permission from 82, copyright 2018 Ivyspring International Publisher.

Recent strategies to modulate TME by PDT have been implemented to enhance the anti-tumor effect of nanomedicine and overcome tumor heterogeneity. Various NPs for PDT have showed a synergistic tumor-targeting effect with chemotherapy against malignant cancer 81. For example, recently developed PDT-utilizing NPs enhanced the anti-cancer effects of FDA-approved cancer nanomedicines by effectively depleting the MDR of cancer cells and increasing their tumor penetration 82. These results are important because current outcomes of conventional nanomedicines relying solely on the EPR effect have been limited due to unsatisfactory cellular internalization of nanomedicine and reduced drug efflux; all FDA-approved nano-drugs are substrates of multidrug-resistance P-glycoprotein (Pgp) (**Figure 5A**) 82. Combined therapy with PDT not only can destroy resistant cancer cells but also enhances the tumor penetration of nanomedicines (**Figure 5B**). As a result, PDT enhances the efficacy of nanomedicine, which results from reduced interstitial pressure, destroyed biological barriers, and the following increased the EPR effect (**Figure 5C**). Similarly, synergistic effect resulting from boosted drug release in PDT combined with chemotherapy shows promise as an alternative avenue to achieve the enhanced EPR effect. Disassembly of NPs by PDT at the tumor site boosted cytotoxic drug release, thus activating a cascade of chemotherapeutic effects and then destroying tumor cells in a drug-resistant tumor model 83. This study implies that PDT can provide a way to overcome critical biological barriers to nanomedicine delivery, which cannot be done with nanomedicine alone. Yan *et al.* recently developed nano-photosensitizers (isophthalic acid/layered double hydroxide nanohybrids) that can show superior cytotoxic properties with a remarkable IC_50_ (approximately 0.1 μg/mL) and safety. Its high safety with superior activity may enable clinical translation allied with the stronger EPR effect 84. The supramolecular photosensitizers take advantage of the NIR laser (808 nm) based markable tissue penetration and exhibit a strong ability to ablate tumors by laser irradiation *in vivo*. Taking recent research into consideration, PDT-utilizing NPs with the EPR effect would provided effective approaches for clinical translation of cancer nanomedicines in cancer therapy.

### 3.2. Combination with SDT

SDT is a emerging non-invasive approach for cancer therapy through activating sonosensitizers by low-energy ultrasound. Sonosensitzers at tumor sites are triggered by ultrasound and then generate reactive oxygen species (ROS) for cancer therapy. SDT has been considered a desirable option for combination with nanomedicine to treat cancers. SDT has sufficient tissue-penetrating depth compared to light in PDT, which is preferable for treating deep-seated tumors and improving the EPR effect of nanomedicine. In addition, SDT can reduce their side effects on normal cells and tissues by its site-specific targeting effect, which can facilitate clinical translation. Over the years, several strategies combining nanomedicine and SDT have been developed in combination with the EPR effect to enhance the therapeutic outcome of anti-cancer therapy. Recent studies show that the EPR effect of sonosensitizers can be improved with a new type of self-assembled nanosonosensitizers. Hangrong Chen *et al*. developed sonosensitizer-containing NPs that facilitate transferrin receptor-mediated endocytosis for deep tissue penetration 85. These NPs intercellularly deliver sonosensitizers protoporphyrin IX through transferrin mediation, overcoming the tissue barrier and improving the EPR effect. In addition, high-intensity focused ultrasound (HIFU) can also be used to enhance the EPR effect of nanomedicine, utilizing non-ionizing ultrasonic waves to induce hyperthermia within target tissue 86. Hyperthermia has been reported to increase the extravasation of NPs into the tumor tissues without toxicity in mice 87. HIFU has been evaluated in various preclinical studies and has been recognized to be relatively close to clinical translation. In view of the above, there are several preclinical tests of SDT in combination with nanomedicine in progress, addressing delivery issue of the sonosensitizers 88. Therefore, considering the evidences of therapeutic effects in SDT with the EPR effect, the combination approach with nanomedicine and SDT for cancer treatment would be promising.

### 3.3. Combination with RT

RT is one of the most commonly used treatments for cancer, either as a monotherapy or in combination with other treatments. RT has formed the mainstream of cancer treatment because it is cost-effective, target-specific and highly effective. Notably, RT is a remarkable therapeutic modality for cancer that is not limited by tissue penetration, destroying tumor cells and the TME. However, although external irradiation can directly kill cancer cells through ROS and energy, RT still has many inevitable shortcomings as single treatment, including dose limitations, severe toxicity and RT resistance 89. Recently, advances in nanotechnology utilizing the EPR effect have enabled novel strategies for nanomedicine treatment in combination with RT 90. In fact, RT significantly increases vascular permeability, which enhances the extravasation of subsequently administered nanomedicine from the vessels and its accumulation in tumor sites 91. A recent study examining the effect of TME-targeted NPs (with RGD) in combination with RT has shown that radiation influences endothelial cells and blood vessels and enhances the therapeutic response to subsequent chemotherapy 92. Clinically, RT is usually administered at low fractionated doses (1.8-3 Gy), sometimes supplemented with a boost dose at the beginning. It has been reported that low doses of RT are able to induce NP accumulation by destroying immature blood vessels. Therefore, the increased vessel permeability by RT has great potential to improve the delivery of nanomedicine via the EPR even in heterogeneous tumors.

Great advances in nanotechnology have substantially diversified the use of PDT, SDT and RT in combination with nanomedicine. Improvement of the EPR effect with PDT, SDT, and RT with the subsequent administration of nanomedicine for chemotherapy, immunotherapy, or RNA interference therapy has synergistically promoted anti-cancer activity 93. Because these therapeutic modalities have shown effectiveness, excellent safety and minimal invasiveness, they are considered to have the great potential to enter into the mainstream of cancer therapy. Alternations to the TME by PDT, SDT, and RT would contribute to overcoming the barriers to the delivery of nanomedicine, which would facilitate successful clinical translation. In a different way with these three methods, magnetic NPs can be efficiently accumulated at tumor tissue by improving the EPR effect. Since magnetic NPs have been clinically in use as a magnetic resonance imaging (MRI) contrast agent from the 1990s, magnetic NPs have been extensively investigated for their clinical applications as DDS 94, 95. Unfortunately, PDT, SDT and RT have been revealed a lack of selectivity of their sensitizers. Therefore, the selective delivery of sensitizing agents into TME using NPs would be optimal option for reducing off-target toxicity and enhancing therapeutic outcome. Overall, these combination strategies incorporating the help of external sources may provide a way to reassess therapeutic agents previously evaluated as suboptimal.

## 4. Physiological remodeling of TME

One of the major challenges that current nanomedicine has yet to address is how to predict the therapeutic efficacy or delivery efficacy of a nanomedicine without understanding the complicated and heterogeneous TME. Multiple studies have demonstrated that the physiological remodeling of TME substantially improves the delivery of drugs to the tumor site. Current strategies for remodeling TME have focused on biologically changing the structural properties of the tumor and its environment to increase nanomedicine delivery to the target sites 12, 96. In this section, we discuss promising TME remodeling approaches to improve the EPR effect: the induction of artificial TME and vascular remodeling.

### 4.1. Induction of artificial TME

**Figure 6:**
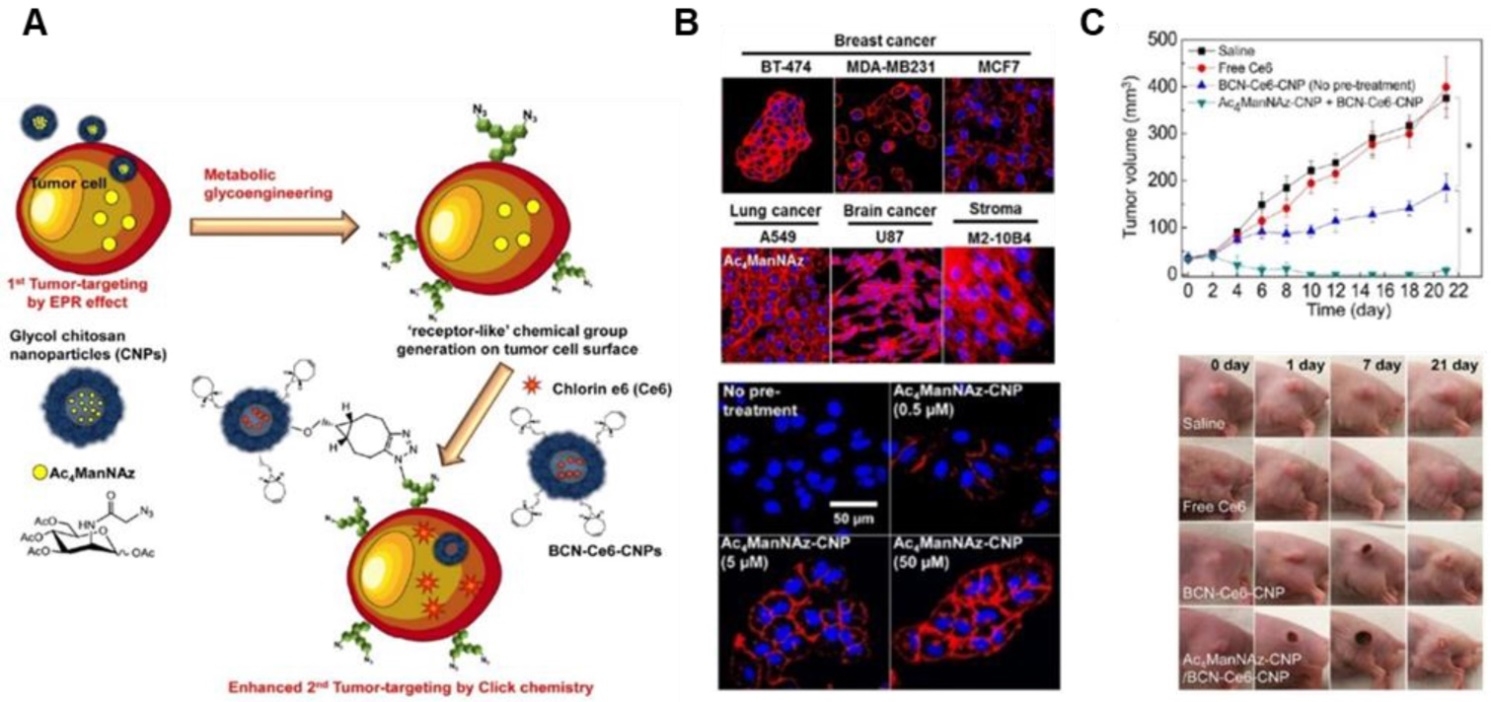
The EPR effect-aided tumor targeting based on metabolic glycoengineering and click chemistry. (A) Schematic illustration of artificial reporter-targeting strategy and mechanism. Adapted with permission from 98, copyright 2014 American Chemical Society. (B) Confocal microscopy images of the generation of azide groups in various Ac_4_ManNAz-treated cells. Adapted with permission from 99, copyright 2018 Elsevier. (C) Tumor inhibitory effect of chitosan nanoparticle (CNP)-based NPs with Ce6 for PDT in A549 tumor-bearing mice and tumor images. Adapted with permission from 98, copyright 2014 American Chemical Society.

The heterogeneity of tumors and the complexity of TME have often presented obstacles even to nanomedicine designed to bind TME-specific molecular markers. Various subpopulations of tumor cells express different kinds and amounts of natural receptors in the TME. To overcome the heterogeneity and complexity of TME, several researchers introduced artificial chemical receptors that are exogenously generated on tumor cells, regardless of phenotypes of tumor cells. In this way, genetically different and heterogeneous tumor cells can be converted into phenotypically uniform cells, leading to a uniform EPR effect for subsequently administered nanomedicine (**Figure 6A**) 97, 98. Previous studies have shown that an artificial azide reporter, originating from the metabolic precursor, tetraacetylated N-azidoacetyl-D-mannosamine (Ac_4_ManNAz) can be presented on the surface of tumor cells by metabolic glycoengineering (**Figure 6B**) 99, 100. Subsequently, Chlorin e6 (Ce6)-containing NPs which are decorated with the ligands, bicycle[6.1.0]nonyne to bind to artificial receptors were administered and bound to artificial receptors on tumors through bioorthogonal click reaction between azide groups and the ligands, leading to photodynamic therapy *in vivo* regardless of tumor types (**Figure 6C**) 98. To generate tumor-specific artificial receptors on the tumor cell surface, peptides responsive to tumor-associated enzymes such as cathepsin B or caspase-3/7 were incorporated into metabolic precursor 101. Peptides responsive to tumor-associated enzymes allow selective induction of artificial receptors on tumor cells. This approach introduced “receptor-like” chemical groups on the surface of tumor cells regardless of tumor types instead of utilizing natural receptors for tumor targeting, demonstrating a way to overcome the heterogeneity of the EPR effect and to improve the EPR effect.

**Figure 7:**
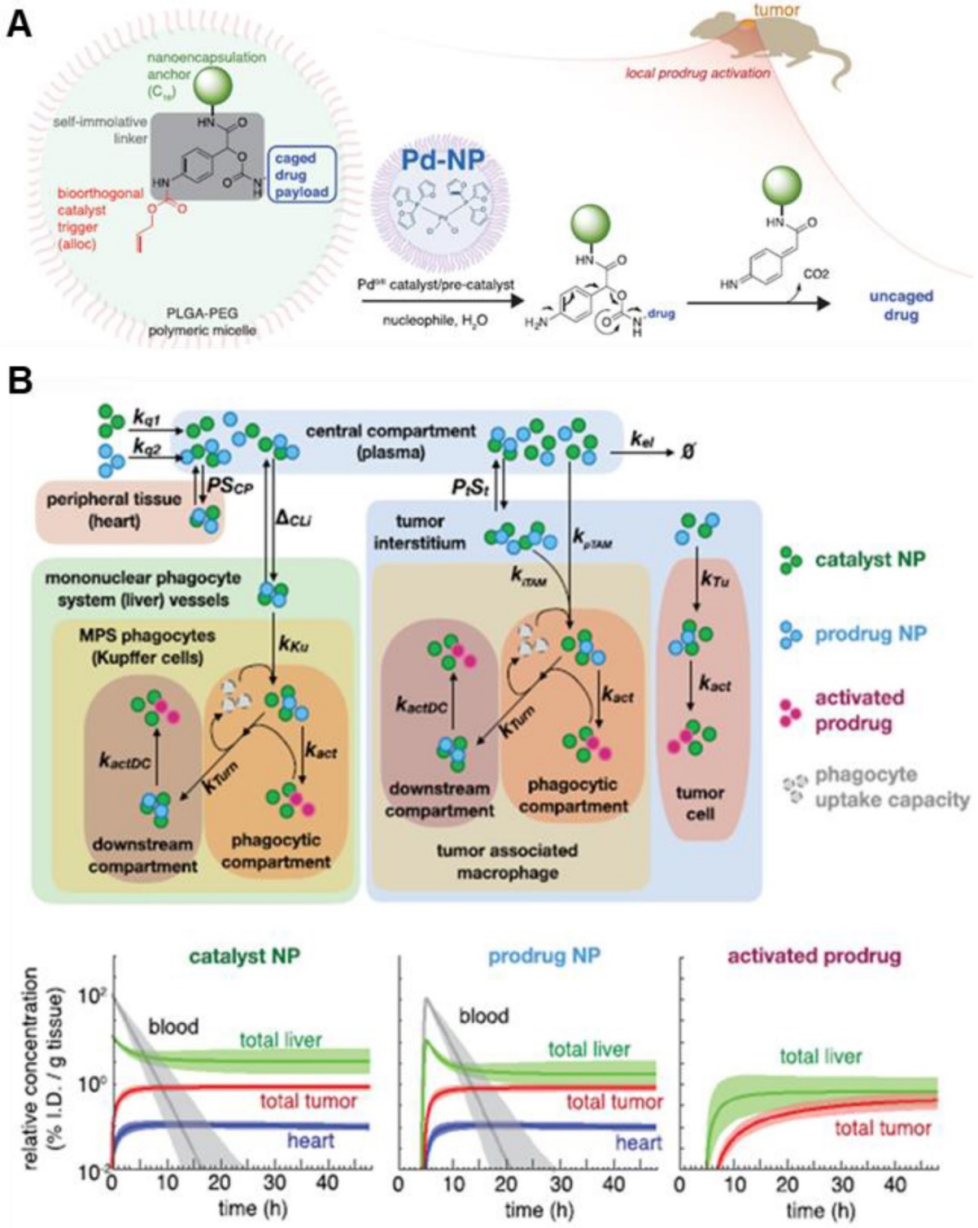
Schematic illustration of NP with bioorthogonal catalyst trigger (allyloxycarbonyl; alloc), prodrug and Pd-based NP (A) The cleavable protective group (alloc) can be removed by a triggering agent (Pd catalyst) when Pd NPs are delivered to the local tumor site by the EPR effect. (B) The calculated computational multicompartment pharmacokinetic model for the catalyst and prodrug NPs show that biodistribution of NPs and activated prodrug over 48 h. Analysis of the results of simulating the use of a combination of catalyst and prodrug NPs by the EPR effect indicates increased selectivity of drug activation in the tumor. Adapted with permission from 78, copyright 2018 American Chemical Society.

In addition to artificial receptors, artificial ECM was constructed to inhibit tumor invasion and metastasis using laminin-mimic peptide-based NPs 102. Laminin, high-molecular weight protein of the basal lamina, is one of the most significant components of ECM, influencing cell differentiation and adhesion. Laminin-mimic peptide-based NPs accumulated in the tumor site due to the EPR effect and transformed into ECM around the tumor, which significantly inhibited lung metastasis in melanoma and breast tumor models. This study is somewhat different from the other studies discussed in this review. While other studies sought to enhance drug delivery to tumors by altering ECM, this study sought to inhibit the movement of tumor cells by altering ECM. Recently, Weissleder *et al*. reported that a palladium catalyst encapsulated in NP (Nano-palladium) demonstrated efficacious catalytic activity *in vivo* in animal models 78. Change of *in vivo* catalytic activity in the tumor sites can be regarded as creating artificial TME for enhanced drug efficacy. Nano-palladium accumulates in solid tumors due to the EPR effect and then activates a DOX prodrug at the tumor site of the tumor model **(Figure 7A)**. Furthermore, a computational multicompartment model of prodrug activation showed the biodistribution of NPs, prodrug, and activated prodrug over 48 h (**Figure 7B**).

Transforming growth factor beta (TGF-β) has played an important role in the conventional TME remodeling, regulating angiogenesis 103 and inhibiting the expansion of T cells 104. Remodeling of the TME by inhibiting TGF-β enables NPs to effectively penetrate the targeted tumor tissue. Kano *et al*. reported that low-doses of TGF-β inhibitor successfully altered the TME including tumor vasculature, which increased the EPR effect with minimal side effects 105. Additionally, TGF-β inhibition in pericyte-abundant BxPC-3 pancreatic cancer vessels increased the uptake of NPs, logically implying the augmented EPR effect 106, 107. The role of other TME-related factors, such as fibroblast growth factor (FGF) and platelet-derived growth factor (PDGF) is under evaluation in terms of TME-remodeling for anti-cancer therapy.

### 4.2. Vascular remodeling

The delivery of EPR-dependent nanomedicine strongly depends on the characteristic of the tumor vasculature, which directly affects the “permeation” of nanomedicine. Therefore, vascular remodeling using DDS has attracted great attention over the last three decades 11, 108. In general, highly pro-angiogenic tumors result in low pericyte coverage and loose cell junctions, leading to leaky and disorganized blood vessels in TME 109. In turn, it reconstructs highly permeable immature vasculature and the elevated interstitial fluid pressure at the tumor site, lead to a high EPR effect 110, 111. At this present, tumor vessel modulation could be an effective way to influence the intensity of the EPR effect. Approaches to remodel the tumor vasculature using anti-angiogenic agents or external stimuli clearly alter in the function of vessels, affecting permeation and disruption 112. In addition, there are approaches that enlarge the endothelial pores which serve as the gateway for nanomedicine to extravasate into TME, by using vasodilators and vasoactive cytokines, such as nitric oxide (NO), nitroglycerine, tumor necrosis factor-alpha (TNF-α), angiotensin-Ⅱ, etc. The effect of the leaky vasculature is discussed in Section 3.1. Xu *et al*., for instance, reported that NO-releasing NPs combining a photosensitizer (IR780) and chemotherapeutic drug (PTX) significantly suppressed tumor growth by boosting tumor vascular permeability 113.

There are many approaches to inhibit new blood vessel formation, not only reducing the supply of oxygen and nutrients to tumor, but also leading to the regression of established tumors 114. The normalization of tumor blood vessels in TME can be achieved by the administration of anti-vascular endothelial growth factor (VEGF) antibodies or VEGF receptor inhibitors 115, 116. It has also been reported that vascular stabilization and maturation are strongly influenced by tyrosine kinase with immunoglobulin-like and EGF-like domains 2 (Tie2) 117. Among FDA-approved therapeutic antibodies against cancer, trastuzumab (marketed as Herceptin^®^) could improve vascular perfusion for the retention of nanomedicines at the tumor site 118, 119. Among these anti-angiogenic therapies, VEGF inhibition has taken center stage due to the significance of the correlation between VEGF and angiogenesis in cancer. Anti-VEGF therapy induces synergistic anti-tumor effects through normalization of tumor vessels 120, 121. VEGF inhibition using siRNA improves the efficacy of concurrent chemotherapy, which may be attributed to enhanced permeation of chemotherapeutic drug-containing NPs 122. On the other hand, vascular-disrupting agents (VDAs) have been used to induce the rapid collapse of tumor vasculature, resulting in vascular remodeling 123. Additionally, appropriate levels of angiogenesis *i.e.*, a certain density and even distribution of tumor blood vessels, practically increase total blood volume and improve perfusion, resulting in enhanced drug accumulation in tumors 124, 125. This alternative approach which is distinct from vascular normalization is called vascular promotion, improves the efficacy of co-administered therapeutics, such as chemotherapeutic drug-containing NPs 126 and anti-PD-1 antibodies 127. This physical change in the TME helps to generate microvascular networks and normal vessels in ECM, contributing to the improvement of the EPR effect 128. Another interesting strategy for improving vascular remodeling is to use anti-hypertensive agents such as losartan, an inhibitor of angiotensin. It has been reported that targeting angiotensin signaling with an angiotensin inhibitor can diminish the ECM, especially collagen and hyaluronan 129, 130. Recently, Zhao *et al.* reported that losartan reduced the physical forces actively exerted by tumor and stromal cells to compress tumor blood vessels in ovarian carcinoma xenograft models. This reduction leads to enhanced delivery and efficacy of the chemotherapeutic drug, PTX via improving vascular perfusion 131. Losartan combined with chemotherapeutic drugs is undergoing clinical trial (National Clinical Trial [NCT] identifier; NCT01821729) for pancreatic tumor treatment. Overall, these diverse approaches to modifying the tumor vasculature mainly affect the permeation of nanomedicine, improving the EPR effect.

The induction of artificial TME, including artificial receptors and ECM, can provide a promising opportunity for improved therapeutic outcomes by neutralizing the inherent heterogeneity of tumor and adding extrinsic homogeneity to the tumor. These approaches can be further expanded to provide a tool for personalized medicine to match the specific need of individual tumors. Although this review does not include immunotherapy, nanomedicine can be combined with immunotherapy agents to overcome the drug resistance and boost the immune response by remodeling the immune cell composition in the TME. For example, remodeling of tumor stroma composed of cancer-associated fibroblasts (CAFs), altering anti-tumor immune response 132 and deleting immune cells to protect tumors 133, has recently come into the spotlight. DDS needs a strategy for tearing down the 'walls' of CAFs to improve the EPR effect and immunotherapy. The CAFs affect the functional polarization of TAMs, which can phagocytize NPs and are thus indirectly able to enhance drug delivery and accumulation 134. Consequently, the EPR effect is improved by increased macrophage infiltration into the TME 135. TAMs are one of the most abundant immune cell populations in the TME and are critical modulators of the TME that directly affect vascularization and ECM remodeling 136. TAMs directly affect tumor vascularization by secreting pro-antigenic factors and directly affect ECM remodeling by releasing ECM-degrading enzymes or by stimulating collagen secretion. These factors make TAMs an attractive target for increasing the EPR effect in cancer treatment. For example, cyclic tumor homing peptide iRGD (CCRGDKGPDC)-based NPs with a macrophage-specific sequence (AAN) could inhibit tumor growth and modulate TME with depletion of TAM 137. In this study, a significant improvement in anti-tumor efficacy and NPs accumulation was achieved by interaction with tumor vascular endothelial cells, indicating the increased EPR effect. The macrophages could be polarized into TAMs, pro-tumorigenic M2 macrophages or anti-tumorigenic M1 macrophages depending on signals in the surrounding environment 138. Selective delivery into TAMs or M1 macrophages is necessary to remodel TME to favor cancer treatment. Zhu *et al*. created PEGylated cowpea mosaic virus particles that could be internalized by TAMs but not M1 macrophages. They suggested that these NPs can be loaded with cytotoxic agents that target the population of TAMs only, causing the population of M1 to rise as the population of TAMs decreases. In addition, as phagocytic inflammatory cells, macrophages are commonly known to be able to phagocytose particles, thereby inducing off-target effects of nanomedicines 139. Davis *et al.* proved that the cellular uptake of TAMs was approximately 3.5-fold greater than that of LKB498 melanoma cancer cells by radiolabeling 140. TAMs could reduce the uptake of NPs by cancer cells in the TME and TAM depletion could thus decrease the off-target uptake of NPs. Therefore, TME modulation by TAM depletion is able to indirectly enhance the EPR effect *in vivo*. Taken together, it increasingly becomes evident that modern immunotherapy can greatly benefit from the expansion of our understanding of nanotechnology and DDS.

## 5. Conclusions and perspectives

Passive targeting strategies based on the EPR effect have shown great therapeutic potential in various preclinical animal models. However, the therapeutic outcome of passively targeted nanomedicine in clinical practice is heterogeneous mainly due to the inherent heterogeneity of the EPR effect. Additionally, the low delivery efficiency of NPs to a solid tumor (approximately 1-5%) indicates that the EPR effect itself may have fundamental limitations for clinical application 141. For the translation of NPs from animal studies to the clinic, various factors such as tumor size, type, and location must be considered carefully 10, 142. In fact, the EPR effect was reported to be maximized in tumors that contain low intratumoral ECM and large amounts of angiogenic blood vessels. This hurdle causes to the limited number of nanomedicines in the current markets since Doxil^®^ (liposomal DOX) emerged as the first nanomedicine in 1995. Over the last two decades, several other nano-drugs including Abraxane (NP with albumin-bound PTX) and Onivyde (liposomal irinotecan) successfully entered the market, showing remarkable therapeutic efficacy in patients. To increase the clinical use of nanomedicine, the low clinical efficacy of the EPR effect also needs to be overcome by novel approaches. In this point of view, recent clinical studies show that novel NPs based strategies enable the design of tumor-specific nanocarriers that recognize biological targets in TME. For example, anti-EGFR-immunoliposomes loaded with DOX have shown high efficacy and low toxicity in phase I and II clinical trials (NCT02833766) for the patients with advanced triple-negative and EGFR-positive breast cancer. Also, ThermoDox which is a heat-activated liposome of DOX, coupled with radiofrequency-induced heating, is in phase III clinical trials for the treatment of hepatocellular carcinoma (NCT02112656) 143. Taken together, these approaches have shown promises for clinical translation of nanomedicine, overcoming the limitations of the EPR effect alone to treat solid tumors such as pancreatic and breast cancer.

In this review, we focused on the current attempts at overcoming the limitations of traditional EPR-dependent nanomedicine by combining with supplementary strategies, such as additional molecular targeting, physical alteration, or physiological remodeling of the TME. The diverse attempts discussed in this review present both limitations and promise for clinical use. To overcome these limitations, further studies are necessary. NPs that are designed to bind TME-specific molecular markers can become more impactful with studies that identify new crucial targets in tumor ECM. In turn, the new findings will lead to innovative nanomedicine platforms that are suitable for the new targets. Additionally, as our understanding of the complex TME and heterogeneity of tumors expands, nanomedicine with multiple complementary targets may prove more beneficial for precise localization of drugs in the body and therapeutic outcomes in the clinic. If the tumor site is externally accessible, another favorable approach is local delivery combined with PDT and SDT to improve the EPR effect, especially in tumors with a low initial EPR effect. Furthermore, inducing the expression of an artificial receptor on the surface of heterogeneous tumor cells can provide an alternative opportunity for the better therapeutic outcome by improving the EPR effect and by adding extrinsic homogeneity to the tumor. The induction of artificial receptors can further be personalized to match the specific needs of individual tumors.

We propose that additional strategies applied in combination with the EPR effect should address the specific characteristics of the TME in various cancers. These additional approaches might greatly advance to the current treatment options for solid tumors (such as colon, breast and pancreatic cancer) with low EPR, overcoming the current limitation of its clinical application of nanomedicine. In this regard, assessing the EPR effect in individuals is crucial for improved therapeutic effects. Clinically available technology for imaging the EPR effect in patients, such as computed tomography, MRI could provide clinicians with valuable information for medication regimens and treatment planning, thus paving the way for personalized nanomedicine 144, 145. Individuals with tumors that exhibit high EPR would be treated with EPR-dependent nanomedicine, while combination treatment in addition to traditional nanomedicine would be applied to the individuals with tumors that produce a low EPR effect. Unfortunately, the current status of EPR imaging is at the developmental stage; thus far, few studies have assessed or clinically analyzed the EPR effect in patients. However, the rapid ongoing developments in nanotechnology, the call for more personalized treatments, and the concurrent expansion of the additional strategies available in conjunction with traditional nanomedicine discussed in this review will doubtlessly raise the number of clinically approved nanomedicines and extend their benefits to more patients in need.

## Figures and Tables

**Table 1:** Recent advances in synergistically combined strategies to improve the EPR effect in TME

Class	Type	Target	Material(composition)	Brief description	Tumor model	Ref
Utilization of TME-specific molecular markers	ECM	CD44 receptor	HA	Thermosensitive self-assembled NPs with HA/PTX	4T1	42
	ECM	EGFR	HA	Dual-targeting strategy with low toxicity	HCCLM3	44
	Enzyme	Cathepsin B	Peptide (FRRG)	Self-assembling carrier-free NPs of prodrug containing DOX	HT-29	66
	Enzyme	MMP-2	Liposome with sodium bicarbonate	Nanoscale micelle systems binding EGFR/HER2 complex	4T1	64
Alteration of TME aided by external sources	PDT	Pgp	Doxil and Abraxane	Depleting MDR cancer cells by PDT using APCs and Doxil^®^	KB, 3T3	82
	PDT	Light-induced ^1^O_2_	Ce6, thioketal linker	ROS-responsive Ce6/DOX-loaded RHPPE NPs for PDT	MCF-7/ADR	83
	PDT	Light-induced ^1^O_2_	RTP/LDH nanohybrids	NIR activated supramolecular photosensitizers for two-photon PDT	HeLa	84
	SDT	Transferrin receptor	Protoporphyrin IX	Nanosonosensitizers for ROS-mediated SDT	HeLa	85
	RT	α_v_β_3_ integrin	AuNPs, cRGD	Sequential chemotherapy after RT using vascular-targeted AuNPs	Sarcoma	92
Physiological remodeling of TME	ECM	Cathepsin B	Peptide (KGRR)	Metabolic precursor for tumor-specific fluorescence imaging	HT-29	101
	Enzyme	Caspase-3/-7	Peptide (KGDEVD)	Metabolic precursor for tumor bioorthogonal apoptosis tracking	PC-3	146
	Vascular	GSH	NO	NO therapy together with IR780 and PTX-loaded NPs	4T1	113
	Vascular	VEGF	PolysiRNA	Combination treatment with metronomic DOX and RNA interference NPs	PC-3	122
	Vascular	Tubulin	Vascular disrupting agent (CKD-516)	Ischemia and necrosis inducing VDA in combination with DOX	VX2	147
	Vascular	RhoA/ROCK	lysophosphatidic acid receptor 4	Vascular network formation for chemo- and immunotherapy	GL261	127
	ECM	Integrin	Peptide (RGD and YIGSR)	Transformable artificial ECM for tumor invasion and metastasis	MDA-MB-231	102
Multifunctional strategies targeting TME	Enzyme	Caspase-3	Peptide (DEVD)	Radiation-induced apoptosis-targeted chemotherapy	C3H/HeN	70
	Vascular	VEGF	Anginex and Avastin	Vessel normalization by angiogenesis inhibitor with RT	MA148, B16F10, SCK	148
	ECM	TAMs	DOX and Taxol	Chemotherapy combined with PDT by TME-remodeling TAMs	4T1	135

APCs: antibody-photosensitizer conjugates, AuNPs: gold nanoparticles, GSH: glutathione, HER2: human epidermal growth factor receptor 2, LDH: layered double hydroxide, MMP-2: matrix metalloproteinase-2, RhoA/ROCK: Ras homolog gene family, member A/ Rho-associated protein kinase, RHPPE: SO-responsive PEGylated hyperbranched polyphosphates, RTP: room temperature phosphorescence

## Abbreviations

Ac_4_ManNAz: tetraacetylated N-azidoacetyl-D-mannosamine
CAFs: cancer-associated fibroblasts
Ce6: Chlorin e6
DDS: drug delivery systems
DOX: doxorubicin
EGF: epidermal growth factor
EGFR: epidermal growth factor receptor
EPI: epirubicin
FDA: Food and Drug Administration
HA: hyaluronic acid
HIF-1α: hypoxia-inducible factor-1α
HIFU: high-intensity focused ultrasound
LIFU: low-intensity focused ultrasound
MDR: multi-drug resistance
MRI: magnetic resonance imaging
NIR: near-infrared
PDT: photodynamic therapy
PEG: polyethylene glycol
PEGPH20: PEGylated recombinant human hyaluronidase
PEI: polyethyleneimine
Pgp: P-glycoprotein
PTX: paclitaxel
RGD: Arg-Gly-Asp
ROS: reactive oxygen species
RT: radiation therapy
SDT: sonodynamic therapy
siRNA: small interfering RNA
TAMs: tumor-associated macrophages
TGF-β: Transforming growth factor beta
TNF-α: tumor necrosis factor-alpha
VDAs: vascular-disrupting agents
VEGF: vascular endothelial growth factor
